# Acute Dilatation, Ischemia, and Necrosis of Stomach without Perforation

**DOI:** 10.1155/2013/984594

**Published:** 2013-10-07

**Authors:** Manash Ranjan Sahoo, Anil T. Kumar, Sunil Jaiswal, Siba Narayan Bhujabal

**Affiliations:** Department of Surgery, SCB. Medical College, Cuttack, Odisha 753007, India

## Abstract

Acute gastric dilatation can have multiple etiologies which may lead to ischemia of the stomach. Without proper timely diagnosis and treatment, potentially fatal events such as gastric perforation, haemorrhage, and other serious complications can occur. Here we present a 36-year-old man who came to the casualty with pain abdomen and distension for 2 days. Clinically, abdomen was asymmetrically distended more in the left hypochondrium and epigastrium region. Straight X-ray abdomen showed opacified left hypochondrium with nonspecific gaseous distension of bowel. Exploratory laparotomy revealed dilated stomach with patchy gangrene over lesser curvature and fundic area. About 4 litres of brownish fluid along with semisolid undigested food particles was sucked out (mainly undigested pieces of meat). Limited resection of gangrenous areas and primary repair were done along with feeding jejunostomy. Necrosis of the stomach was confirmed on histopathology. The patient recovered well and was discharged on the tenth postoperative day.

## 1. Introduction

Acute gastric dilatation can have multiple etiologies which may lead to ischemia of the stomach. The etiologies are lifestyle habits, underlying morbidities, acute necrotizing inflammation, acute vascular insufficiency, and postoperative complications. Without proper timely diagnosis and treatment, potentially fatal events such as gastric perforation, haemorrhage, and other serious complications can occur. We here present a rare case of gastric dilatation leading to patchy gangrenes on the surface of stomach and how timely intervention was carried out.

## 2. Case Report

**Figure 1:**
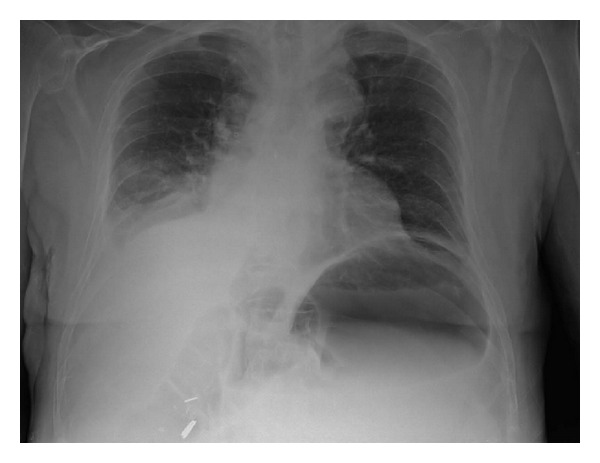
X-ray chest and abdomen showing opacification in the left hypochondrium.

**Figure 2:**
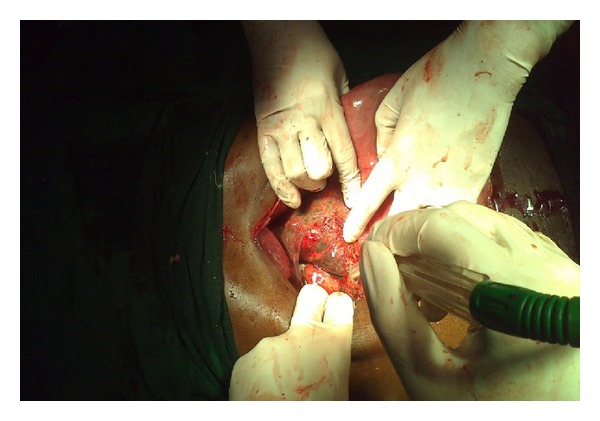
Gangrenous area on lesser curvature with dilated stomach.

**Figure 3:**
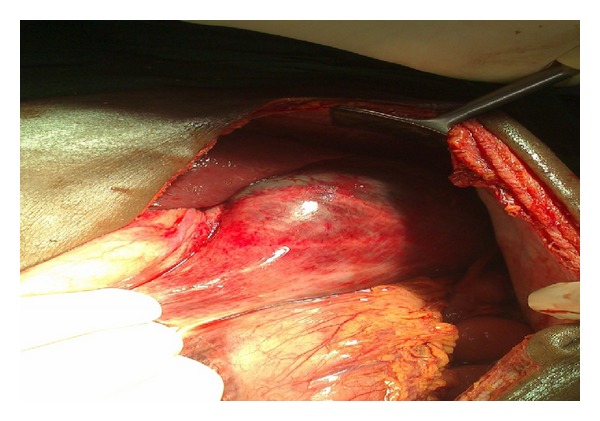
Gangrenous area on the fundus of the stomach.

**Figure 4:**
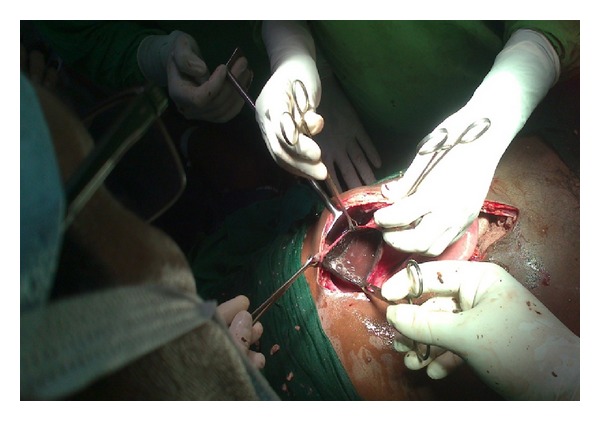
Perforation in lesser curvature gangrenous area after handling of that area.

**Figure 5:**
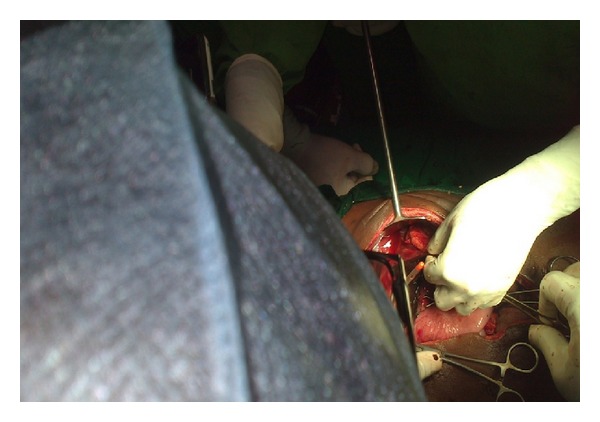
Nasogastric tube tip visible after sucking of semisolid thick brown contents.

**Figure 6:**
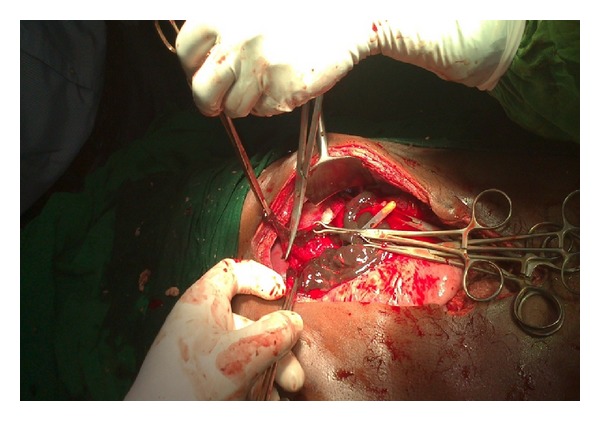
Resection of gangrenous area till fresh bleeding occurs.

**Figure 7:**
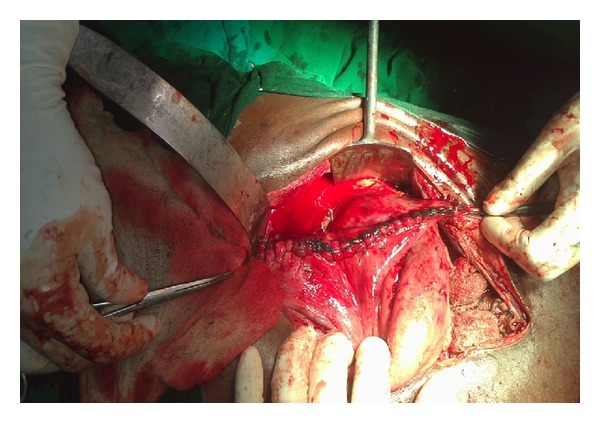
Primary closure of the defect by suturing using 2-0 vicryl.

**Figure 8:**
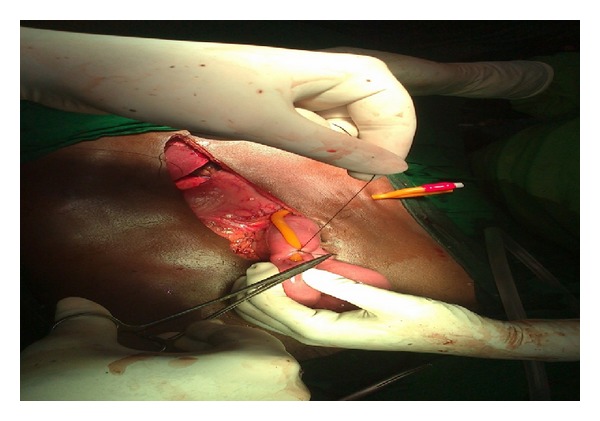
Feeding jejunostomy done.

A 36-year-old male patient, referred from periphery hospital with nasogastric tube in place, presented to the casualty with pain abdomen and abdominal distension for two days which was not relieved with conservative treatment. Two days ago he had taken nonvegetarian meal twice, in increased quantity than usual, within a short gap of 3 hours between those two meals. Then he had two episodes of vomiting 6 hours later. His past history was not significant. He was not suffering from any psychiatric illness or any co-morbidity like diabetes and had not undergone any surgeries. His vital parameters were within normal limits. Abdominal examination showed more asymmetrical distension in left hypochondrium and epigastrium with tympanicity all over the abdomen without signs of peritonitis. Straight X-ray abdomen showed opacified left hypochondrium with nonspecific gaseous distension of bowel (Figure 1). Even after conservative treatment, when the distension and pain did not subside, he was planned for exploratory laparotomy. Ryle's tube aspiration in this case was unproductive. On opening the abdomen through upper midline incision it was found that stomach was dilated with patchy gangrene at two areas, one on the lesser curvature (Figure 2) and the other on the fundus of stomach (Figure 3). Handling at the gangrenous area leads to perforation at the lesser curvature which showed that there was impending perforation in that area. Through this perforation about, 4 litres of thick brown coloured fluid mixed with undigested food particles (mainly undigested pieces of meat) was sucked out (Figure 4) and removed. The tip of the nasogastric tube now became visible through the defect (Figure 5). The gangrenous area was resected till there was fresh bleeding from the margin (Figure 6). Specimen was sent for histopathological examination. The defect thus created was suture repaired primarily in single layer interrupted fashion with vicryl 2-0 suture material (Figure 7). The same was repeated for gangrene of the fundus. Feeding jejunostomy was done (Figure 8). Postoperatively, patient was started with feeds through feeding jejunostomy tube on the 3rd day. He was given liquids orally on the 7th day and semisolid diet on the 8th day and was discharged on the 10th postoperative day. Histopathological examination revealed mucosal ulceration, prominent areas of haemorrhage and edema in submucosa, and thinning of the muscle layer suggestive of necrosis of the resected specimen of the stomach.

## 3. Discussion

In 1833, Duplay first described acute gastric dilatation [1]. Acute ischemic necrosis of stomach is a very rare disease due to its abundant vascular supply. In experimental animals, in order to produce ischemic necrosis, closure of the right and left gastric and gastroepiploic arteries together with at least 80% of the collaterals is required [2]. The important causes are postoperative complications [3, 4], anorexia nervosa and bulimia, psychogenic polyphagia, diabetes mellitus, trauma, electrolyte disturbances, gastric volvulus, and spinal conditions [1, 5–10]. In our case, the patient had binge eating 6 hours before development of symptoms. Since the dilated stomach fully contained undigested semisolid food materials with thick brown coloured fluid, we could attribute this dilatation to binge eating. He did not have any past history of psychiatric illness, diabetic gastropathy, any other comorbid illness, trauma, or any previous surgery on stomach. A thorough search of PubMed revealed only less than 50 cases of acute gastric dilatation, ischemia, and necrosis recorded till now in the literature. 

Ischemia is caused presumably due to venous insufficiency when massive dilatation occurs [11, 12]. To impair venous outflow, either 14 mmHg of pressure or more than 3 litres of fluid is sufficient, although more than 15 litres has been described in eating disorders in chronic distension. Rupture can occur with intragastric pressures of more than 120 mmHg or 4 litres of fluid. In the majority of the cases, greater curvature and gastric fundus are more prone for necrosis and require emergent treatment [13]. Lesser curvature and pyloric regions of the stomach tend to be spared [1]. A consequence in events as postulated by Abdu et al. is mucosal necrosis, followed by full-thickness involvement of the gastric wall and perforation [10–12]. Surgery may be avoided if the diagnosis is established in an early stage. A mortality rate of 80% to 100% has been reported due to gastric ischemia and perforation as a result of dilation [14]. In our case, there was impending perforation at the area of gangrene on lesser curvature which was tackled before it could perforate and lead to devastating complication. Necrosis of the gastric wall was confirmed by histopathology which showed features like mucosal ulceration, prominent areas of haemorrhage and edema in submucosa, and thinning of the muscle layer.

Several theories have been postulated to explain the pathogenesis of acute gastric dilatation. Morris et al. claimed that anaesthesia and debilitation may be predisposing factor as it is a very frequent postoperative complication. Relaxation of the upper oesophageal sphincter with aerophagia may be a factor leading to gastric distention [3, 4, 10]. In 1859, Brinton introduced the atonic theory [10]. The stomach undergoes atony and muscular atrophy during a period of starvation, so that a sudden ingestion of food overtaxes an already weakened stomach in patients with eating disorders. In 1861, von Rokitansky proposed superior mesenteric artery syndrome (mechanical theory) in which vascular compression of the third segment of the duodenum, between superior mesenteric artery, aorta, and vertebral column, causes acute gastric dilatation [5]. Other authors suggest that pancreatitis, peptic ulcer, gallbladder disease, and appendicitis also cause acute gastric dilatation [15, 16] and infectious causes like necrotizing gastritis generally involving immunocompromised patients like diabetes, AIDS, and neoplasia are also reported [17, 18]. 

In more than 90% of cases of acute gastric dilatation, vomiting is an important and common symptom [19]. Another sign reported in the literature is the inability to vomit which is not fully understood. This may be due to the occlusion of the gastroesophageal junction by the distended fundus, which angulates the esophagus against the right crus of the diaphragm, producing a one-way valve [20]. Significant, diffuse abdominal distension accompanied by abdominal pain is common. 

Plain abdominal films and CT scan can demonstrate gastric distension and free air and are useful in the diagnosis. In our case, since the distension was increasing and huge, we just planned to explore the patient with just abdominal X-ray showing opacification in left hpochondrium with gaseous distension of bowel. Treatment focuses on early diagnosis and decompression of the stomach, thus halting the vascular congestion and thus ischemia [21]. Decompression with nasogastric tube should be the first step in the management, followed by immediate surgery in case of perforation. A normal size nasogastric tube may prove to be inefficient in decompressing stomach. Sometimes, when semisolid material is present in the stomach, even a large tube may be inefficient. In our case too since the contents were semisolid nasogastric tube was nonproductive. If conservative measures fail or gastric infarction with or without perforation is suspected, immediate surgical intervention is mandatory [10]. Necrosis might be partial or involving the full organ. Total gastrectomy is the procedure of choice [14], but it requires time and stable hemodynamic conditions. Partial resections have already been described in case of patchy necrosis and gangrene [22]. Hence, we did a type of sleeve resection removing only gangrenous area with primary repair with feeding jejunostomy with minimal morbidity to the patient. We justify limited resection rather than gastrectomy as first line of treatment in such cases since stomach is such a vascularised organ; even modified sleeve or partial resection with primary repair will suffice. Surgeons should be aware that acute gastric dilatation may occur even in patients who are not diagnosed as having a typical eating disorder after an episode of binge eating. A high index of suspicion is necessary to diagnose this condition in order to avoid fatal complications. First line of treatment should be conservative with nasogastric decompression. If it fails, necessary timely surgery would prevent unnecessary morbidity. Even though total gastrectomy is the treatment of choice, modified sleeve or partial resection can be done safely in limited patchy necrosis and gangrene.
